# Drug retention of 7 biologics and tofacitinib in biologics-naïve and biologics-switched patients with rheumatoid arthritis: the ANSWER cohort study

**DOI:** 10.1186/s13075-020-02232-w

**Published:** 2020-06-15

**Authors:** Kosuke Ebina, Toru Hirano, Yuichi Maeda, Wataru Yamamoto, Motomu Hashimoto, Koichi Murata, Tohru Takeuchi, Hideyuki Shiba, Yonsu Son, Hideki Amuro, Akira Onishi, Kengo Akashi, Ryota Hara, Masaki Katayama, Keiichi Yamamoto, Atsushi Kumanogoh, Makoto Hirao

**Affiliations:** 1 Department of Musculoskeletal Regenerative Medicine, Graduate School of Medicine, Osaka University, Osaka, Japan; 2 Department of Respiratory Medicine and Clinical Immunology, Graduate School of Medicine, Osaka University, Osaka, Japan; 3 Department of Health Information Management, Kurashiki Sweet Hospital, Okayama, Japan; 4 Department of Advanced Medicine for Rheumatic Diseases, Graduate School of Medicine, Kyoto University, Kyoto, Japan; 5 Department of Internal Medicine (IV), Osaka Medical College, Osaka, Japan; 6 First Department of Internal Medicine, Kansai Medical University, Osaka, Japan; 7 Department of Rheumatology and Clinical Immunology, Kobe University Graduate School of Medicine, Hyogo, Japan; 8 The Center for Rheumatic Diseases, Nara Medical University, Nara, Japan; 9 Department of Rheumatology, Osaka Red Cross Hospital, Osaka, Japan; 10 Department of Medical Informatics, Wakayama Medical University Hospital, Wakayama, Japan; 11 Department of Orthopaedic Surgery, Osaka University, Graduate School of Medicine, Osaka, Japan

**Keywords:** ANSWER cohort, Biological disease-modifying antirheumatic drugs, Drug retention, Rheumatoid arthritis

## Abstract

**Background:**

This multi-center, retrospective study aimed to clarify retention rates and reasons for discontinuation of 7 biological disease-modifying antirheumatic drugs (bDMARDs) and tofacitinib (TOF), one of the janus kinase inhibitors, in bDMARDs-naïve and bDMARDs-switched patients with rheumatoid arthritis (RA).

**Methods:**

This study assessed 3897 patients and 4415 treatment courses with bDMARDs and TOF from 2001 to 2019 (2737 bDMARDs-naïve courses and 1678 bDMARDs-switched courses [59.5% of switched courses were their second agent], female 82.3%, baseline age 57.4 years, disease duration 8.5 years; rheumatoid factor positivity 78.4%; Disease Activity Score in 28 joints using erythrocyte sedimentation rate 4.3; concomitant prednisolone [PSL] dose 6.1 mg/day [usage 42.4%], and methotrexate [MTX] dose 8.5 mg/week [usage 60.9%]). Treatment courses included abatacept (ABT; *n* = 663), adalimumab (ADA; *n* = 536), certolizumab pegol (CZP; *n* = 226), etanercept (ETN; *n* = 856), golimumab (GLM; *n* = 458), infliximab (IFX; *n* = 724), tocilizumab (TCZ; *n* = 851), and TOF (*n* = 101/only bDMARDs-switched cases). Drug discontinuation reasons (categorized into lack of effectiveness, toxic adverse events, non-toxic reasons, or remission) and rates were estimated at 36 months using Gray’s test and statistically evaluated after adjusted by potential clinical confounders (age, sex, disease duration, concomitant PSL and MTX usage, starting date, and number of switched bDMARDs) using the Fine-Gray model.

**Results:**

Cumulative incidence of drug discontinuation for each reason was as follows: lack of effectiveness in the bDMARDs-naïve group (from 13.7% [ABT] to 26.9% [CZP]; *P* < 0.001 between agents) and the bDMARDs-switched group (from 18.9% [TCZ] to 46.1% [CZP]; *P* < 0.001 between agents); toxic adverse events in the bDMARDs-naïve group (from 4.6% [ABT] to 11.2% [ETN]; *P* < 0.001 between agents) and the bDMARDs-switched group (from 5.0% [ETN] to 15.7% [TOF]; *P* = 0.004 between agents); and remission in the bDMARDs-naïve group (from 2.9% [ETN] to 10.0% [IFX]; *P* < 0.001 between agents) and the bDMARDs-switched group (from 1.1% [CZP] to 3.3% [GLM]; *P* = 0.9 between agents).

**Conclusions:**

Remarkable differences were observed in drug retention of 7 bDMARDs and TOF between bDMARDs-naïve and bDMARDs-switched cases.

## Introduction

Tumor necrosis factor inhibitors (TNFi), such as infliximab (IFX), etanercept (ETN), and adalimumab (ADA), were the first biological disease-modifying antirheumatic drugs (bDMARDs) used for rheumatoid arthritis (RA) that had accumulated evidence of drug retention [1–5]. Other TNFi such as golimumab (GLM) (2011) and certolizumab pegol (CZP) (2013), and the first Janus kinase inhibitor (JAKi), tofacitinib (TOF) (2013), were recently approved in Japan. The European League against Rheumatism (EULAR) provided recommendations in 2016 regarding the management of RA with bDMARDs, in which CTLA4-Ig (abatacept [ABT]), anti-interleukin (IL)-6 receptor antibody (tocilizumab [TCZ]), and JAKi are considered equivalent to TNFi [6]. They also mentioned that there is no difference in outcomes among these bDMARDs and JAKi, irrespective of their mechanism of action. Moreover, Smolen et al. reported that these bDMARDs have similar efficacy in previously TNFi-experienced patients, although efficacy may decrease compared with bDMARDs-naïve patients [7]. However, cohort-based studies revealed that in patients who showed inadequate response to TNFi, switching to a non-TNFi agent (such as ABT, rituximab, or TCZ) showed significantly higher drug retention rates compared with switching to another TNFi [8, 9]. Taken together, it is evident that these drug retention (reflecting both safety and effectiveness) may differ between bDMARDs-naïve and bDMARDs-switched cases.

Randomized controlled trials (RCTs) often recruit patients with fewer comorbidities who are different from those in real-world settings [10], and cohort-based observational studies have increasingly been used to investigate the performance of bDMARDs [1–4, 9, 11, 12]. In particular, drug retention is considered as a major index of both safety and effectiveness [4, 13–15]. To the best of our knowledge, there are no reports comparing drug retention rates of 7 bDMARDs and TOF, especially in both bDMARDs-naïve and bDMARDs-switched cases.

We recently reported drug retention rates among bDMARDs used in all age [16, 17] as well as among the elderly population [18], factors associated with the achievement of bDMARDs-free remission [19], and the correlation of treatment response with family history of RA [20] from our cohort. Since then, we are continuously accumulating new data. The aim of this multi-center, retrospective study was to clarify the retention rates of 7 bDMARDs and TOF in both bDMARDs-naïve and bDMARDs-switched cases in a real-world setting.

## Materials and methods

### Patients

The Kansai Consortium for Well-being of Rheumatic Disease Patients (ANSWER) cohort is an observational multi-center registry of patients with RA in the Kansai district of Japan. Data from RA patients who fulfilled the 1987 RA classification criteria of the American College of Rheumatology [21] or the 2010 ACR/EULAR RA classification criteria [22] at 6 universities and one university-affiliated hospital (Kyoto University, Osaka University, Osaka Medical College, Kansai Medical University, Kobe University, Nara Medical University, and Osaka Red Cross Hospital) were included [23]. In this study, patients who were newly treated with at least 1 of 7 bDMARDs (ABT, ADA, CZP, ETN, GLM, IFX, and TCZ; including both intravenous and subcutaneous agents, but excluding biosimilar agents) or TOF from 2001 to 2019, with data on starting and discontinuation dates and reasons for discontinuation, were included. In addition, baseline demographic data such as age, sex, duration of disease, disease activity (Disease Activity Score in 28 joints using erythrocyte sedimentation rate [DAS28-ESR]), clinical disease activity index (CDAI), number of previously administered bDMARDs, concomitant doses and ratio of methotrexate (MTX) and prednisolone (PSL) (other glucocorticoids were calculated as equivalent dose to PSL; MTX or PSL dose was not considered when agents were not combined), rheumatoid factor (RF) and anti-cyclic citrullinated peptide antibody (ACPA) positivity, and Health Assessment Questionnaire [HAQ] disability index [DI] score were also collected [16–18].

Treatments were administered by the attending rheumatologists in accordance with the guidelines of the Japan College of Rheumatology [24–26]. The starting date of each biologic was classified into 3 groups: 2001–2009, 2010–2013, and 2014–2019, according to the released date [IFX (2003), ETN (2005), ADA (2008), TCZ (2008), ABT (2010), GLM (2011), CZP (2013), TOF (2013), sarilumab (2017), baricitinib (2017), peficitinib (2019), and ETN biosimilar (2019) (some of them were used as investigational agents before commercially released)] to equalize the released agents’ number and possible influence of other agents on physicians’ prescription decision in each duration. Drug retention was retrospectively evaluated as the duration until definitive treatment interruption. Reasons for discontinuation were analyzed and classified into 4 major categories: (1) lack of effectiveness (including primary and secondary); (2) toxic adverse events (infection, skin or systemic reaction, and other toxic events, including hematologic, pulmonary, renal, cardiovascular complications, and malignancies, etc.); (3) non-toxic reasons (patient preference, change in hospital, desire for pregnancy, etc.); and (4) disease remission [16–18]. Physicians were allowed to cite only one reason for discontinuation. Then, treatment cases were separated into bDMARDs-naïve cases (without TOF) and bDMARDs-switched cases (all cases of TOF were switched from bDMARDs).

### Statistical analysis

The estimated cumulative incidence curves and discontinuation ratio of each agent defined by specific reasons at 36 months were examined by Gray’s test [27, 28]. The discontinuation ratio of the agents at 36 months was analyzed and statistically compared using the Fine-Gray hazard competing risk regression model [27, 28], adjusted by potential confounders that may influence drug retention as previously described (age, sex, disease duration, concomitant PSL and MTX usage, starting date, and number of switched bDMARDs) [1, 9, 11, 12, 29]. Statistical analyses were performed using EZR (Saitama Medical Center, Jichi Medical University, Saitama, Japan), a graphical user interface for R (The R Foundation for Statistical Computing, Vienna, Austria) [30]. *P* < 0.05 was considered statistically significant.

## Results

### Baseline characteristics

Baseline clinical characteristics of the bDMARDs-naïve cases are shown in Table 1. Overall, mean age was 57.0 years, 81.8% of participants were female, mean disease duration was 7.3 years, RF positivity was 78.6%, ACPA positivity was 81.4%, mean DAS28-ESR score was 4.4, mean CDAI was 17.8, and mean HAQ-DI score was 1.0. Mean doses and ratio of concomitant medications were PSL 6.3 mg/day (39.6%) and MTX 8.6 mg/week (65.4%).

**Table 1:** Clinical characteristics at initiation of 7 bDMARDs (bDMARDs-naïve cases)

Variable	ABT (*n* = 390)	ADA (*n* = 374)	CZP (*n* = 135)	ETN (*n* = 616)	GLM (*n* = 208)	IFX (*n* = 650)	TCZ (*n* = 364)
Age (years)	65.5 ± 12.4	55.3 ± 12.8	58.1 ± 16.8	55.5 ± 15.9	62.0 ± 14.8	52.9 ± 13.4	56.6 ± 14.4
Female sex (%)	81.2	79.4	88.8	86.7	86.1	78.0	78.2
Disease duration (years)	9.2 ± 12.4	5.0 ± 7.5	4.7 ± 7.6	8.3 ± 8.7	7.3 ± 10.0	6.9 ± 8.4	7.4 ± 9.4
RF positivity (%)	86.6	73.8	86.2	83.1	75.6	74.2	74.0
ACPA positivity (%)	84.3	75.9	85.7	83.2	73.2	82.9	82.1
DAS28-ESR	4.4 ± 1.2	4.1 ± 1.2	4.6 ± 1.4	4.4 ± 1.4	4.3 ± 1.2	4.5 ± 1.6	4.6 ± 1.5
CDAI	17.7 ± 9.6	14.7 ± 9.1	22.2 ± 12.9	17.3 ± 8.8	17.2 ± 11.5	18.5 ± 12.4	18.1 ± 9.8
HAQ-DI	1.2 ± 0.8	0.7 ± 0.6	1.2 ± 0.8	0.9 ± 0.8	1.1 ± 0.8	1.1 ± 0.9	1.1 ± 0.8
PSL usage (%)	44.2	32.8	44.8	39.2	38.9	36.4	45.7
PSL dose (mg/day)	3.1 ± 7.3	2.9 ± 4.9	1.7 ± 2.6	2.8 ± 3.6	2.3 ± 3.6	3.1 ± 5.9	2.8 ± 3.9
MTX usage (%)	49.1	72.0	76.1	39.4	76.0	100.0	51.2
MTX dose (mg/week)	8.1 ± 2.8	9.4 ± 3.1	9.4 ± 2.9	8.0 ± 2.8	9.2 ± 2.9	8.2 ± 2.5	8.8 ± 3.0
Starting date 2001–2009 (%)	0.0	13.6	0.0	40.4	1.0	60.9	11.5
Starting date 2010–2013 (%)	35.9	53.7	12.6	42.0	40.4	30.2	47.3
Starting date 2014–2019 (%)	64.1	32.7	87.4	17.6	58.7	8.9	41.2

Values are mean ± standard deviation or percentages. *bDMARDs* biological disease-modifying antirheumatic drugs, *ABT* abatacept, *ADA* adalimumab, *CZP* certolizumab pegol, *ETN* etanercept, *GLM* golimumab, *IFX* infliximab, *TCZ* tocilizumab, *RF* rheumatoid factor, *ACPA* anti-cyclic citrullinated peptide antibody, *DAS28-ESR* Disease Activity Score in 28 joints using erythrocyte sedimentation rate, *CDAI* clinical disease activity index, *HAQ-DI* Health Assessment Questionnaire disability index, *PSL* prednisolone, *MTX* methotrexate

Baseline clinical characteristics of the bDMARDs-switched cases are shown in Table 2. Overall, mean age was 58.1 years, 83.3% of participants were female, mean disease duration was 10.5 years, RF positivity was 78.1%, ACPA positivity was 83.4%, mean DAS28-ESR score was 4.2, mean CDAI was 15.7, and mean HAQ-DI score was 1.1. Mean doses and ratio of concomitant medications were PSL 5.7 mg/day (49.3%) and MTX 8.3 mg/week (57.1%). The bDMARDs were administered as the second agent in 59.5% of patients and as the third or latter agent in 40.5% of patients.

**Table 2:** Clinical characteristics at initiation of 7 bDMARDs and tofacitinib (bDMARDs-switched cases)

Variable	ABT (*n* = 273)	ADA (*n* = 162)	CZP (*n* = 91)	ETN (*n* = 240)	GLM (*n* = 250)	IFX (*n* = 74)	TCZ (*n* = 487)	TOF (*n* = 101)
Age (years)	61.5 ± 13.2	55.4 ± 14.8	54.1 ± 15.4	55.5 ± 15.7	60.5 ± 14.6	53.5 ± 12.6	58.1 ± 14.1	59.7 ± 13.6
Female sex (%)	81.3	87.7	85.7	82.1	88.0	79.5	82.5	77.2
Disease duration (years)	11.2 ± 9.3	9.7 ± 9.0	9.9 ± 9.0	9.4 ± 8.1	12.0 ± 10.2	10.9 ± 16.0	10.0 ± 8.9	11.0 ± 8.6
RF positivity (%)	77.8	78.2	77.2	75.6	78.1	72.7	79.9	80.0
ACPA positivity (%)	84.4	80.9	84.8	86.5	82.9	82.4	83.3	73.3
DAS28-ESR	4.3 ± 1.3	3.9 ± 1.1	4.4 ± 1.5	4.1 ± 1.4	4.0 ± 1.4	4.0 ± 1.6	4.4 ± 1.4	4.3 ± 1.3
CDAI	14.7 ± 9.5	11.9 ± 8.8	16.3 ± 10.8	13.7 ± 10.0	14.6 ± 10.2	18.9 ± 13.0	16.3 ± 10.3	19.3 ± 11.3
HAQ-DI	1.1 ± 0.8	0.8 ± 0.7	1.2 ± 0.9	0.9 ± 0.8	1.1 ± 0.8	1.0 ± 1.0	1.2 ± 0.8	1.0 ± 0.8
PSL usage (%)	55.1	44.1	40.7	47.0	46.0	42.5	52.2	54.5
PSL dose (mg/day)	6.4 ± 4.1	5.9 ± 4.3	4.9 ± 2.9	5.6 ± 3.8	5.1 ± 3.5	5.6 ± 3.1	6.1 ± 3.9	4.1 ± 3.1
MTX usage (%)	47.8	57.1	62.6	50.0	66.1	100.0	54.9	51.5
MTX dose (mg/week)	8.4 ± 3.0	8.0 ± 2.9	8.4 ± 3.1	8.3 ± 2.7	8.0 ± 3.1	8.7 ± 2.8	8.4 ± 3.1	9.0 ± 3.3
Starting date 2001–2009 (%)	0.0	25.3	0.0	27.5	0.0	20.3	10.9	0.0
Starting date 2010–2013 (%)	43.2	49.4	26.4	37.5	48.8	51.4	45.8	2.0
Starting date 2014–2019 (%)	56.8	25.3	73.6	35.0	51.2	28.4	43.3	98.0
2nd bio or TOF (%)	54.6	75.9	41.8	74.6	58.8	70.3	57.3	31.7
≥ 3rd bio or TOF (%)	45.4	24.1	58.2	25.4	41.2	29.7	42.7	68.3

Values are mean ± standard deviation or percentages. *bDMARDs* biological disease-modifying antirheumatic drugs, *ABT* abatacept, *ADA* adalimumab, *CZP* certolizumab pegol, *ETN* etanercept, *GLM* golimumab, *IFX* infliximab, *TCZ* tocilizumab, *TOF* tofacitinib, *RF* rheumatoid factor, *ACPA* anti-cyclic citrullinated peptide antibody, *DAS28-ESR* Disease Activity Score in 28 joints using erythrocyte sedimentation rate, *CDAI* clinical disease activity index, *HAQ-DI* Health Assessment Questionnaire disability index, *PSL* prednisolone, *MTX* methotrexate, *bio* biologic agent

### Drug retention and causes for discontinuation

**Fig. 1:**
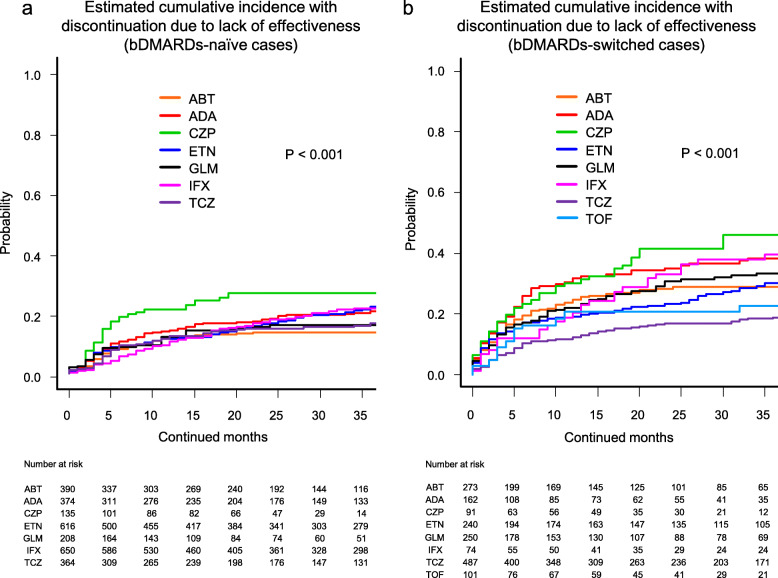
Estimated cumulative incidence with discontinuation due to lack of effectiveness in the bDMARDs-naïve cases (**a**) and the bDMARDs-switched cases (**b**). ABT abatacept, ADA adalimumab, CZP certolizumab pegol, ETN etanercept, GLM golimumab, IFX infliximab, TCZ tocilizumab, TOF tofacitinib, bDMARDs biological disease-modifying antirheumatic drugs

**Fig. 2:**
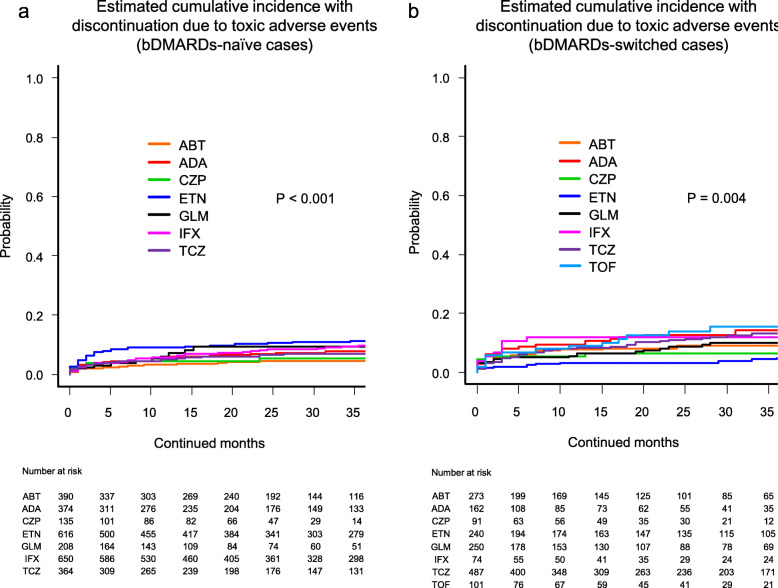
Estimated cumulative incidence with discontinuation due to toxic adverse events in the bDMARDs-naïve cases (**a**) and the bDMARDs-switched cases (**b**). ABT abatacept, ADA adalimumab, CZP certolizumab pegol, ETN etanercept, GLM golimumab, IFX infliximab, TCZ tocilizumab, TOF tofacitinib, bDMARDs biological disease-modifying antirheumatic drugs

**Fig. 3:**
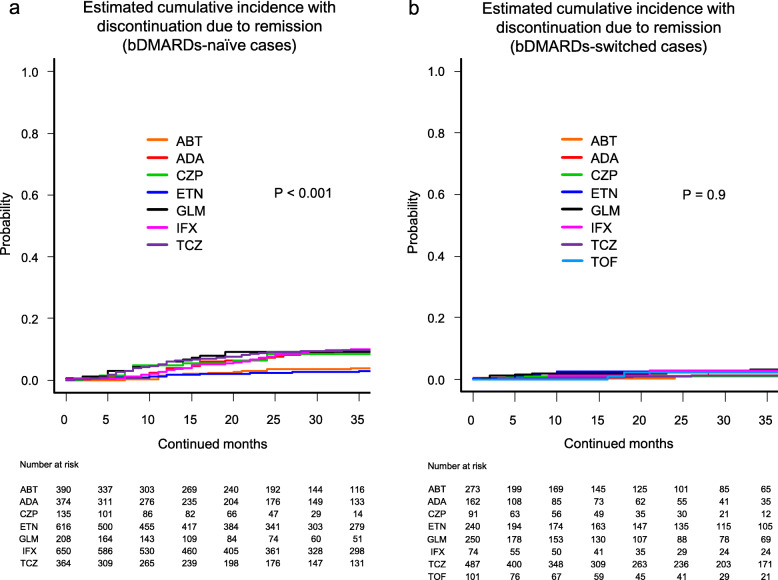
Estimated cumulative incidence with discontinuation due to remission in the bDMARDs-naïve cases (**a**) and the bDMARDs-switched cases (**b**). ABT abatacept, ADA adalimumab, CZP certolizumab pegol, ETN etanercept, GLM golimumab, IFX infliximab, TCZ tocilizumab, TOF tofacitinib, bDMARDs biological disease-modifying antirheumatic drugs

**Fig. 4:**
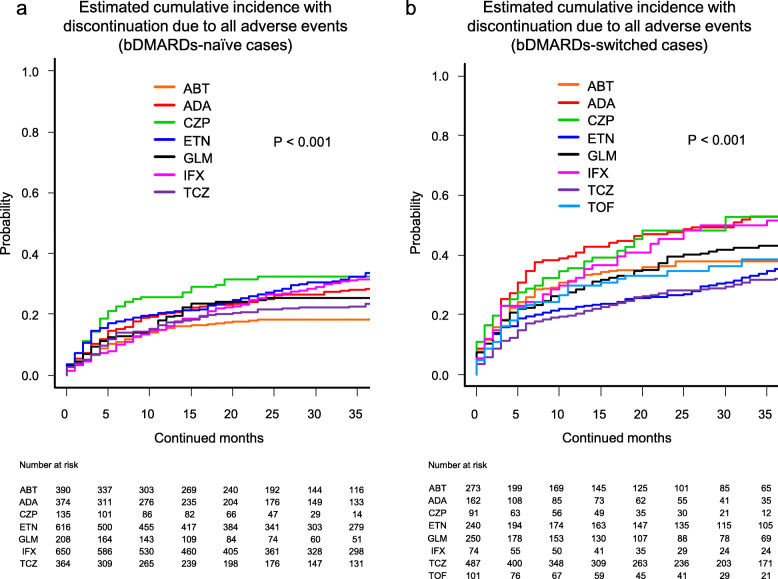
Estimated cumulative incidence with discontinuation due to all adverse events (including lack of effectiveness and toxic adverse events) in the bDMARDs-naïve cases (**a**) and the bDMARDs-switched cases (**b**). ABT abatacept, ADA adalimumab, CZP certolizumab pegol, ETN etanercept, GLM golimumab, IFX infliximab, TCZ tocilizumab, TOF tofacitinib, bDMARDs biological disease-modifying antirheumatic drugs

Cause-specific cumulative discontinuation rates were assessed using Gray’s test and statistically compared using Fine-Gray hazard competing risk regression model at 36 months (Figs. 1, 2, 3, and 4 and Supplementary Fig. 1).

Drug discontinuation rates due to lack of effectiveness in the bDMARDs-naïve cases were as follows (Fig. 1a): ABT (13.7%), GLM (16.1%), TCZ (16.6%), ADA (20.6%), IFX (21.8%), ETN (22.4%), and CZP (26.9%) (*P* < 0.001). These rates in the bDMARDs-switched cases were as follows (Fig. 1b): TCZ (18.9%), TOF (22.8%), ABT (28.7%), ETN (30.3%), GLM (33.3%), ADA (38.4%), IFX (39.4%), and CZP (46.1%) (*P* < 0.001).

Drug discontinuation rates due to toxic adverse events in the bDMARDs-naïve cases were as follows (Fig. 2a): ABT (4.6%), CZP (5.5%), TCZ (6.8%), ADA (7.9%), GLM (9.3%), IFX (9.7%), and ETN (11.2%) (*P* < 0.001). These rates in the bDMARDs-switched cases were as follows (Fig. 2b): ETN (5.0%), CZP (6.6%), ABT (9.2%), GLM (9.9%), IFX (12.2%), TCZ (13.2%), ADA (14.3%), and TOF (15.7%) (*P* = 0.004).

Drug discontinuation rates due to remission in the bDMARDs-naïve cases were as follows (Fig. 3a): ETN (2.9%), ABT (4.0%), CZP (8.4%), GLM (9.0%), ADA (9.8%), TCZ (9.8%), and IFX (10.0%) (*P* < 0.001). These rates in the bDMARDs-switched cases were as follows (Fig. 3b): CZP (1.1%), TCZ (1.2%), ABT (1.4%), ADA (2.1%), TOF (2.3%), ETN (2.5%), IFX (2.8%), and GLM (3.3%) (*P* = 0.9).

Drug discontinuation rates due to non-toxic events in the bDMARDs-naïve cases were as follows (Supplementary Fig. 1a): CZP (3.8%), IFX (9.0%), ABT (10.8%), TCZ (11.4%), ADA (12.3%), ETN (13.5%), and GLM (17.2%) (*P* = 0.07). These rates in the bDMARDs-switched cases were as follows (Supplementary Fig. 1b): CZP (4.1%), GLM (7.0%), ETN (7.4%), TOF (7.7%), IFX (8.5%), TCZ (9.4%), ABT (11.3%), and ADA (14.6%) (*P* = 0.5).

Finally, drug discontinuation rates due to all adverse events (including lack of effectiveness and toxic adverse events) in the bDMARDs-naïve cases were as follows (Fig. 4a): ABT (18.3%), TCZ (23.5%), GLM (25.3%), ADA (28.4%), IFX (31.5%), CZP (32.4%), and ETN (33.6%) (*P* < 0.001). These rates in the bDMARDs-switched cases were as follows (Fig. 4b): TCZ (32.1%), ETN (35.2%), ABT (37.9%), TOF (38.5%), GLM (43.2%), IFX (51.6%), ADA (52.7%), and CZP (52.7%) (*P* < 0.001).

Hazard ratios (HRs) and 95% confidence intervals (CI) for discontinuation due to each specific cause were calculated using the Fine-Gray hazard competing risk regression model adjusted for confounders (Tables 3 and 4).

**Table 3:** Hazard ratio of treatment discontinuation in the bDMARDs-naïve cases (Fine-Gray hazard competing risk regression model, adjusted by baseline age, sex, disease duration, concomitant PSL and MTX usage, and starting date of bDMARDs)

	Reference	HR (95% CI)						
Variable	ABT (*n* = 390)	ADA (*n* = 374)	CZP (*n* = 135)	ETN (*n* = 616)	GLM (*n* = 208)	IFX (*n* = 650)	TCZ (*n* = 364)	*P* value
Lack of effectiveness	1	1.4 (1.0–2.1)	2.4 (1.5–3.8)***	1.7 (1.2–2.4)**	1.1 (0.7–1.7)	1.5 (1.1–2.2)*	1.1 (0.8–1.7)	< 0.001
All toxic adverse events	1	2.8 (1.5–5.2)***	1.7 (0.7–4.0)	4.0 (2.3–6.9)***	2.5 (1.3–4.8)**	4.3 (2.5–7.3)***	2.2 (1.2–4.2)*	< 0.001
Non-toxic reasons	1	0.8 (0.5–1.3)	0.3 (0.1–0.9)*	1.1 (0.7–1.6)	1.5 (0.9–2.5)	1.0 (0.7–1.5)	1.1 (0.7–1.8)	0.07
Remission	1	2.9 (1.5–5.4)***	1.8 (0.8–4.4)	1.0 (0.5–2.0)	2.4 (1.2–5.0)*	3.1 (1.7–5.6)***	2.5 (1.3–4.8) **	< 0.001
All adverse events (including lack of effectiveness and toxic adverse events)	1	1.8 (1.3–2.5)***	2.5 (1.6–3.7) ***	2.3 (1.7–3.1)***	1.5 (1.0–2.2)*	2.1 (1.6–2.9)***	1.4 (1.0–2.0)*	< 0.001

*bDMARDs* biological disease-modifying antirheumatic drugs, *PSL* prednisolone, *MTX* methotrexate, *HR* hazard ratio, *95% CI* 95% confidence interval, *ABT* abatacept, *ADA* adalimumab, *CZP* certolizumab pegol, *ETN* etanercept, *GLM* golimumab, *IFX* infliximab, *TCZ* tocilizumab

*P < 0.05, **P < 0.01, ***P < 0.001

**Table 4:** Hazard ratio of treatment discontinuation in the bDMARDs-switched cases (Fine-Gray hazard competing risk regression model, adjusted by baseline age, sex, disease duration, concomitant PSL and MTX usage, starting date, and number of switched bDMARDs)

	Reference	HR (95% CI)							
Variable	ABT (*n* = 273)	ADA (*n* = 162)	CZP (*n* = 91)	ETN (*n* = 240)	GLM (*n* = 250)	IFX (*n* = 74)	TCZ (*n* = 487)	TOF (*n* = 101)	*P* value
Lack of effectiveness	1	1.3 (0.9–1.8)	1.5 (1.0–2.2)*	1.1 (0.8–1.5)	1.0 (0.7–1.3)	1.3 (0.9–2.0)	0.6 (0.4–0.8)***	0.8 (0.5–1.2)	< 0.001
All toxic adverse events	1	1.8 (1.0–3.1)	0.8 (0.3–2.0)	0.4 (0.2–0.9)*	1.0 (0.6–1.9)	1.2 (0.5–2.7)	1.4 (0.9–2.3)	1.8 (0.9–3.5)	0.004
Non-toxic reasons	1	1.2 (0.6–2.2)	0.3 (0.1–1.1)	0.8 (0.4–1.4)	0.8 (0.4–1.5)	0.9 (0.4–2.4)	0.8 (0.5–1.3)	0.6 (0.2–1.5)	0.5
Remission	1	0.8 (0.1–5.0)	0.9 (0.1–9.2)	1.4 (0.3–6.1)	1.8 (0.4–7.7)	1.9 (0.4–10.7)	1.5 (0.4–5.4)	2.3 (0.4–13.8)	0.9
All adverse events (including lack of effectiveness and toxic adverse events)	1	2.7 (1.6–4.3)***	2.2 (1.4–3.4)**	1.2 (0.8–2.0)	1.4 (1.0–2.1)	2.0 (1.0–3.7)*	0.9 (0.6–1.4)	1.1 (0.6–1.9)	< 0.001

*bDMARDs* biological disease-modifying antirheumatic drugs, *PSL* prednisolone, *MTX* methotrexate, *HR* hazard ratio, *95% CI* 95% confidence interval, *ABT* abatacept, *ADA* adalimumab, *CZP* certolizumab pegol, *ETN* etanercept, *GLM* golimumab, *IFX* infliximab, *TCZ* tocilizumab, *TOF* tofacitinib

*P < 0.05, **P < 0.01, ***P < 0.001

In the bDMARDs-naïve cases (Table 3), HRs for discontinuation due to lack of effectiveness were significantly higher with CZP (HR = 2.4, *P* < 0.001), ETN (HR = 1.7, *P* < 0.01), and IFX (HR = 1.5, *P* < 0.05) compared with ABT (*P* < 0.001 between agents). In terms of toxic adverse events, ADA (HR = 2.8, *P* < 0.001), ETN (HR = 4.0, *P* < 0.001), GLM (HR = 2.5, *P* < 0.01), IFX (HR = 4.3, *P* < 0.001), and TCZ (HR = 2.2, *P* < 0.05) showed a significantly higher rate compared with ABT (*P* < 0.001 between agents). HR for discontinuation due to non-toxic reasons was significantly lower with CZP (HR = 0.3, *P* < 0.05) compared with ABT, although no significant difference was observed between agents (*P* = 0.07). HRs for discontinuation due to remission were significantly higher with ADA (HR = 2.9, *P* < 0.001), GLM (HR = 2.4, *P* < 0.05), IFX (HR = 3.1, *P* < 0.001), and TCZ (HR = 2.5, *P* < 0.01) compared with ABT (*P* < 0.001 between agents). Finally, HRs for all adverse events (including lack of effectiveness and toxic adverse events) were significantly higher with ADA (HR = 1.8, *P* < 0.001), CZP (HR = 2.5, *P* < 0.001), ETN (HR = 2.3, *P* < 0.001), GLM (HR = 1.5, *P* < 0.05), IFX (HR = 2.1, *P* < 0.001), and TCZ (HR = 1.4, *P* < 0.05) compared with ABT (*P* < 0.001 between agents).

In the bDMARDs-switched cases (Table 4), HRs for discontinuation due to lack of effectiveness were significantly higher with CZP (HR = 1.5, *P* < 0.05), although significantly lower with TCZ (HR = 0.6, *P* < 0.001) compared with ABT (*P* < 0.001 between agents). As for all toxic adverse events, ETN (HR = 0.4, *P* < 0.05) showed a significantly lower rate compared with ABT (*P* = 0.004 between agents). There were no significant differences in HRs for discontinuation due to non-toxic reasons (*P* = 0.5) and remission (*P* = 0.9) between agents. Finally, HRs for all adverse events (including lack of effectiveness and toxic adverse events) were significantly higher with ADA (HR = 2.7, *P* < 0.001), CZP (HR = 2.2, *P* < 0.01), and IFX (HR = 2.0, *P* < 0.05) compared with ABT (*P* < 0.001 between agents).

## Discussion

This multi-center, retrospective study was designed to evaluate retention rates and reasons for discontinuation for 7 bDMARDs and TOF, especially in bDMARDs-naïve and bDMARDs-switched cases.

Factors affecting bDMARD retention rates have been reported. Higher age [3], female sex [5], concomitant PSL [3], high DAS28 or HAQ scores [3, 11, 31], absence or low dose of combined MTX [3, 11], and the number of previously used bDMARDs [11] were negative predictors of retention rates in previous studies. With reference to these previous reports, we selected age, sex, disease duration, concomitant PSL and MTX, starting date, and number of switched bDMARDs as adjustment confounders [16–18].

In terms of toxic adverse events, 2016 EULAR recommendations concluded that there were no differences in serious infections or malignancies across bDMARDs [32]. However, cohort-based studies revealed that among TNFi, ETN showed a lower rate of adverse events compared with IFX [3, 5] and ADA [3]. Another report showed that toxic adverse events such as lupus-like events and vasculitis-like events tended to be lowest with CZP compared with other TNFi [33]. In terms of non-TNFi, ABT showed a lower risk of hospitalized infection rates compared with all other bDMARDs [34], and possible increased safety of ABT compared with other agents in RA-associated interstitial lung disease is also reported [35].

Regarding total retention of TNFi, GLM showed a higher retention rate compared with other TNFi when clinical backgrounds were matched [36]. On the other hand, previous studies showed that ETN showed a higher total retention rate compared with ADA and IFX [3, 5]. With respect to differences between TNFi and non-TNFi agents, Jones et al. reported that ABT or TCZ showed higher retention rates compared with TNFi [37]. Moreover, we previously reported that TCZ showed a higher retention rate compared with ADA and IFX [38], and both ABT and TCZ showed higher retention compared with TNFi [16, 17].

Patients with first TNFi failure, switching to non-TNFi bDMARDs showed higher retention rates due to lack of effectiveness compared with patients switched to a second TNFi [9]. In such cases, both ABT and TCZ resulted in substantial improvement in clinical disease activity [39] along with good retention rates [40]. In terms of a JAK inhibitor, TOF showed a lower discontinuation rate due to lack of efficacy and an equivalent rate of adverse events compared with ABT, GLM, and TCZ [41]. However, another report demonstrated that TCZ showed the highest clinical response in such cases, followed by ABT or TOF [42]. Taken together, among the TNFi, ETN and GLM may show good retention, and in bDMARDs-switched cases, non-TNFi such as ABT, TCZ, and TOF may show good retention compared to TNFi. These results are comparable to this study, although discontinuation rate of ETN due to toxic adverse events was relatively high in bDMARDs-naïve cases (especially within 5 months). Considering patients’ background, patients who were treated by ETN as first bDMARDs were combined with relatively low rate of MTX (39.4%), which may suggest the existence of comorbidities leading to MTX intolerance and high rate of toxic adverse events. Interestingly, there were remarkable differences between bDMARDs-naïve and bDMARDs-switched cases in terms of drug retention in this study. Most of the agents’ retention due to lack of effectiveness decreased in bDMARDs-switched cases compared with bDMARDs-naïve cases, although TCZ and ETN showed similar retention rates.

The efficacy of low-dose MTX in Japanese populations compared with western populations should be mentioned. Intraerythrocyte MTX-polyglutamate concentrations, which are considered a useful biomarker of MTX efficacy, were 65 nmol/L with 13.4 mg/week of MTX in the USA, compared with 94 nmol/L with 10.3 mg/week of MTX in Japanese [43]. Thus, a relatively low dose of MTX may have positive effects on bDMARD retention in Japanese populations.

Some limitations to this study need to be considered. First, the backgrounds of patients differed between agents, which may affect results even after adjustment for potential confounders (e.g., MTX may strongly affect the retention of TNFi compared to that of non-TNFi); in addition, comorbidities that may affect drug retention could not be evaluated. Second, the judgment and reasons for discontinuation (such as lack of effectiveness or remission) depended on the decisions of each physician, without standardized criteria. Third, the difference between intravenous and subcutaneous bDMARDs and the use of other conventional synthetic DMARDs could not be determined. Fourth, dose changes of bDMARDs, MTX, and PSL could not be monitored. Fifth, among agents available in Japan, CZP and TOF were licensed most recently (2013), which may have led to a small number of prescriptions (i.e., we could not collect enough data for TOF in bDMARDs-naïve cases), which may have affected results. However, the strength of this study is that it is the first study comparing drug retention and discontinuation reasons of 7 bDMARDs and TOF between bDMARDs-naïve and bDMARDs-switched cases, based on a real-world setting. These results may provide important evidences for the precision medicine, especially for appropriate use of bDMARDs and TOF in both situations of daily clinical practice.

## Conclusions

Remarkable differences were observed in drug retention rates of 7 bDMARDs and TOF between bDMARDs-naïve and bDMARDs-switched cases. Overall retention rates excluding non-toxic reasons and remission were highest with ABT among the bDMARDs-naïve cases (not including TOF), while TCZ showed the highest total retention rate in the bDMARDs-switched cases.

## Supplementary information

**Additional file 1: Figure S1.** Estimated cumulative incidence with discontinuation due to non-toxic events in the bDMARDs-naïve cases (a) and the bDMARDs-switched cases (b). ABT = abatacept, ADA = adalimumab, CZP = certolizumab pegol, ETN = etanercept, GLM = golimumab, IFX = infliximab, TCZ = tocilizumab, TOF = tofacitinib, bDMARDs = biological disease-modifying antirheumatic drugs.

## Data Availability

The datasets used and/or analyzed in the current study are available from the corresponding author on reasonable request.
