# Supplementary material for: Drug retention of 7 biologics and tofacitinib in biologics-naïve and biologics-switched patients with rheumatoid arthritis: the ANSWER cohort study
Source: Arthritis Res Ther. 2020 Jun 15;22:142. doi: 10.1186/s13075-020-02232-w (PMC7296929; doi:10.1186/s13075-020-02232-w)
Supplement: Supplementary file 1 — Additional file 1: Figure S1. Estimated cumulative incidence with discontinuation due to non-toxic events in the bDMARDs-naïve cases (a) and the bDMARDs-switched cases (b). ABT = abatacept, ADA = adalimumab, CZP = certolizumab pegol, ETN = etanercept, GLM = golimumab, IFX = infliximab, TCZ = tocilizumab, TOF = tofacitinib, bDMARDs = biological disease-modifying antirheumatic drugs. [file 13075_2020_2232_MOESM1_ESM.zip › revise ANSWER naive switch ART supplementary figure legend.docx]

**Supplementary Figure 1.** **Estimated cumulative incidence with discontinuation due to non-toxic events in the bDMARDs-naïve cases (a) and the bDMARDs-switched cases (b).**

ABT = abatacept, ADA = adalimumab, CZP = certolizumab pegol, ETN = etanercept, GLM = golimumab, IFX = infliximab, TCZ = tocilizumab, TOF = tofacitinib, bDMARDs = biological disease-modifying antirheumatic drugs.
