# Supplementary material for: Drug retention of 7 biologics and tofacitinib in biologics-naïve and biologics-switched patients with rheumatoid arthritis: the ANSWER cohort study
Source: Arthritis Res Ther. 2020 Jun 15;22:142. doi: 10.1186/s13075-020-02232-w (PMC7296929; doi:10.1186/s13075-020-02232-w)
Supplement: Supplementary file 1 — Additional file 1: Figure S1. Estimated cumulative incidence with discontinuation due to non-toxic events in the bDMARDs-naïve cases (a) and the bDMARDs-switched cases (b). ABT = abatacept, ADA = adalimumab, CZP = certolizumab pegol, ETN = etanercept, GLM = golimumab, IFX = infliximab, TCZ = tocilizumab, TOF = tofacitinib, bDMARDs = biological disease-modifying antirheumatic drugs. [file 13075_2020_2232_MOESM1_ESM.zip › revise ART naive switch supplementary figure1 no1.pptx]

## Slide 1
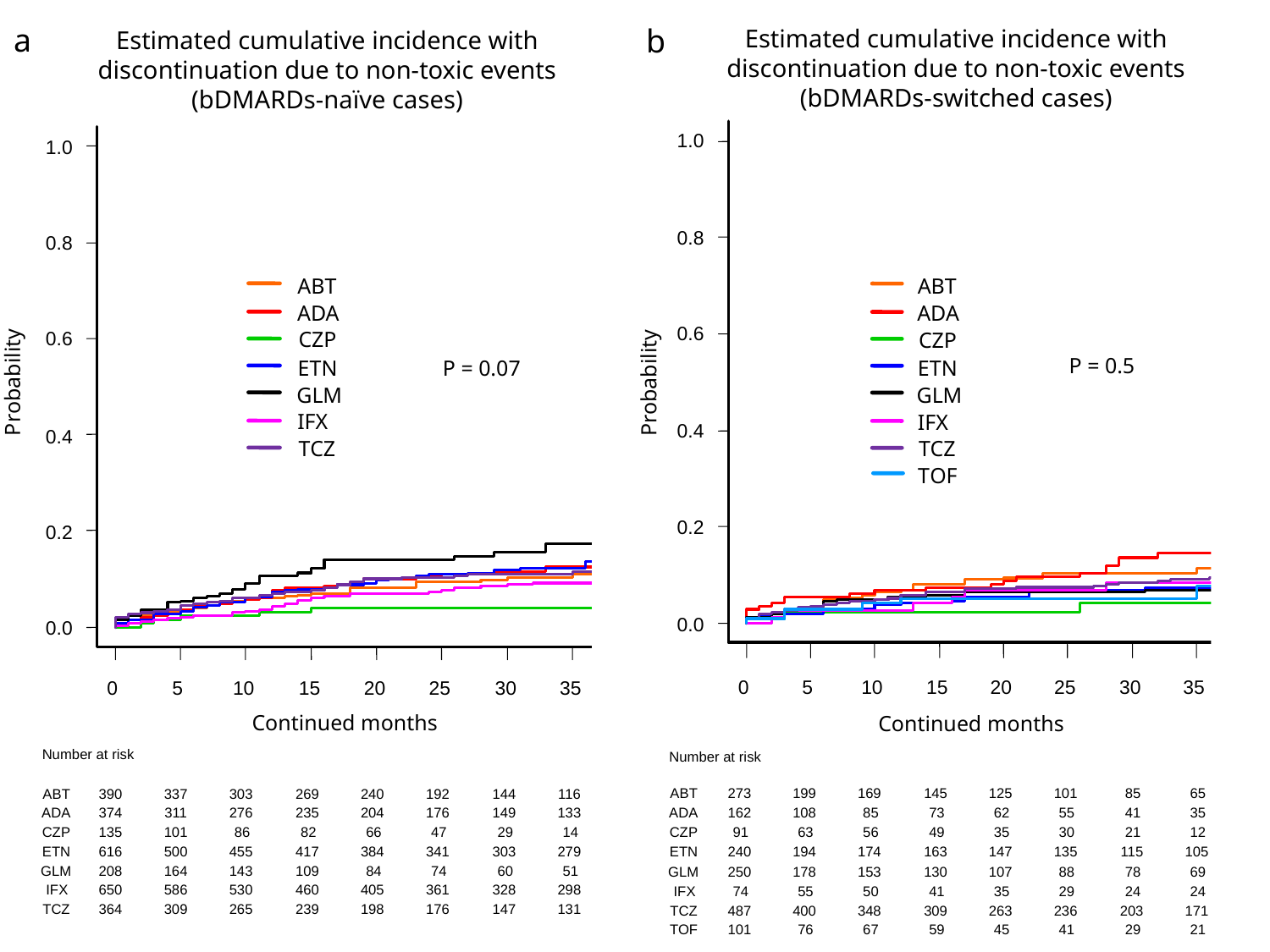

a
b
Estimated cumulative incidence with discontinuation due to non-toxic events
(bDMARDs-switched cases)
Estimated cumulative incidence with discontinuation due to non-toxic events
(bDMARDs-naïve cases)
1.0
0.8
0.6
0.4
0.2
0.0
0
5
10
15
20
25
30
35
1.0
0.8
0.6
0.4
0.2
0.0
0
5
10
15
20
25
30
35
ABT
ADA
CZP
ETN
GLM
IFX
TCZ
ABT
ADA
CZP
ETN
GLM
IFX
TCZ
TOF
P = 0.5
P = 0.07
 Probability
 Probability
Continued months
Continued months
Number at risk
ABT
390
337
303
269
240
192
144
116
ADA
374
311
276
235
204
176
149
133
CZP
135
101
86
82
66
47
29
14
ETN
616
500
455
417
384
341
303
279
GLM
208
164
143
109
84
74
60
51
IFX
650
586
530
460
405
361
328
298
TCZ
364
309
265
239
198
176
147
131
Number at risk
ABT
273
199
169
145
125
101
85
65
ADA
162
108
85
73
62
55
41
35
CZP
91
63
56
49
35
30
21
12
ETN
240
194
174
163
147
135
115
105
GLM
250
178
153
130
107
88
78
69
IFX
74
55
50
41
35
29
24
24
TCZ
487
400
348
309
263
236
203
171
TOF
101
76
67
59
45
41
29
21
